# Health-related quality of life of children and their parents 6 months after children’s critical illness

**DOI:** 10.1007/s11136-019-02347-x

**Published:** 2019-11-06

**Authors:** José Hordijk, Sascha Verbruggen, Ilse Vanhorebeek, Greet Van den Berghe, Elisabeth Utens, Koen Joosten, Karolijn Dulfer

**Affiliations:** 1 Intensive Care Unit, Department of Pediatrics and Pediatric Surgery, Erasmus Medical Centre - Sophia Children’s Hospital, Dr. Molewaterplein 60, 3015 GJ Rotterdam, The Netherlands; 2 Department of Child and Adolescent Psychiatry/Psychology, Erasmus Medical Centre - Sophia Children’s Hospital, Wytemaweg 8, 3015 CN Rotterdam, The Netherlands; 3 Clinical Division and Laboratory of Intensive Care Medicine, Department of Cellular and Molecular Medicine, KU Leuven, Herestraat 49, 3000 Leuven, Belgium; 4 Research Institute of Child Development and Education, University of Amsterdam, Nieuwe Achtergracht 127, 1018 WS Amsterdam, The Netherlands; 5 Academic Center for Child and Adolescent Psychiatry the Bascule, Amsterdam UMC, Academic Medical Centre, Rijksstraatweg 145, 1115 AP Amsterdam, The Netherlands

**Keywords:** PICU, Critical illness, HRQoL, Follow-up

## Abstract

**Purpose:**

This study aimed to examine health-related quality of life (HRQoL) of children and their parents, 6 months after the child’s admission to the Pediatric Intensive Care Unit (PICU). Associations between parents’ reports regarding HRQoL of their child and of themselves were investigated, as well as associations between children’s baseline variables and their parent-reported HRQoL outcomes.

**Methods:**

This is a secondary analysis of cross-sectional data collected in a group of children who participated in the PEPaNIC trial. Six months after discharge from the PICU, parents of critically ill children completed the Infant–Toddler Quality of Life Questionnaire (ITQOL, for age 0–3 years) or the Child Health Questionnaire-Parent Form 50 (CHQ-PF50, for age 4–18 years), which are parallel questionnaires. Parents completed the Short Form Health Survey (SF-12) regarding their own HRQoL. Results were compared with normative data.

**Results:**

At 6 months’ follow-up, 86 children of the 1343 (6%) had died which resulted in 1257 eligible children. Parents of 576 surviving children (46%) completed the questionnaires. Children of responding parents had less often an acute reason for admission and differed in diagnosis compared with children of non-responders. PICU children scored lower on most ITQOL (*n* = 390) scales and CHQ-PF50 (*n* = 186) scales compared with normative data. Parents reported (*n* = 570) higher scores on the physical (*p *< 0.001) and lower scores on the mental SF-12 scale (*p *< 0.001) compared with normative data. Parents̕ mental HRQoL correlated with HRQoL they reported for their child (Pearson Correlations range 0.25–0.57, *p *< 0.001–0.002). Shorter length of stay, lower risk of mortality, younger age, and cardiac diagnosis were associated with higher parent-reported HRQoL outcomes for the child.

**Conclusions:**

Six months after PICU discharge, critically ill children have lower HRQoL compared with normative data. The mental component of HRQoL is impaired in parents and is associated with lower overall parent-reported HRQoL of their child.

**Electronic supplementary material:**

The online version of this article (10.1007/s11136-019-02347-x) contains supplementary material, which is available to authorized users.

## Introduction

Critical illness is known as the dependency on one or more forms of technology to sustain vital functions or the involvement of persistent multiple vital organ system. Children who are critically ill are admitted to a Pediatric Intensive Care Unit (PICU). PICU admissions are highest within the first year of life and respiratory problems are among the most common reasons for admissions at any age [1]. The majority of critically ill children admitted to the PICU recover rapidly with regard to physical functioning [2]. However, a significant proportion is confronted with prolonged consequences that interfere with normal development, such as psychosocial and neurocognitive deficits [3]. The impact of these prolonged consequences on daily life is highly dependent on the individual perception of the patients and their parents. For example, one patient might perceive hearing problems as a burden, but another patient with a similar problem might not feel this is limiting their quality of life. Therefore, patient-reported outcome measures (PROMs) and parent-reports have gained more interest in assessing patients’ health [4]. These PROMs give insight in patients’ subjective evaluation of their health status. A frequently used PROM is Health-Related Quality of Life (HRQoL), which comprises multiple domains, such as physical, psychological, and social wellbeing. In other words, HRQoL reflects the impact of health on the broad concept of quality of life and provides insight in what the impairments mean for the daily life of the patient [5, 6]. Previous studies in PICU survivors showed that critical illness affects HRQoL after discharge, with lower HRQoL scores 1 to 9 months after critical illness than those of healthy children [7, 8].

Young children are not able to reliably evaluate their own HRQoL. Therefore, parents or caregivers usually assess HRQoL of their child, which is called the proxy-report. Parents, who are usually the primary caregivers, are thought to have the most reliable information of the child since they are closely involved in the child’s life. Previous studies in parents focused on specific psychological symptoms such as post-traumatic stress, depression, and anxiety [3]. However, studies investigating the relationship between parents’ own HRQoL on their proxy-reports are scarce.

Previous studies that examined HRQoL in children who were admitted to the PICU used small sample sizes and focused on groups of patients with a specific diagnosis [9, 10]. The present study assessed HRQoL for a large, heterogeneous cohort of children aged 0 to 18 years old, 6 months after critical illness and studied the relationship with parents’ HRQoL. The heterogeneity of the cohort adds value to the generalizability of the results to the general population of critically ill children. Insight in the subjective health status of the critically ill child after PICU admission could lead to early identification of impairments and prevention of delays in the development of the child [2]. Furthermore, analyzing the characteristics of children who particularly have an impaired HRQoL makes it possible to determine which children will benefit from follow-up interventions.

The aims of the current study were therefore threefold. The first aim was to examine HRQoL of children and their parents 6 months after critical illness of the child compared to normative data. The hypothesis was to find lower parent-reported HRQoL scores in critically ill children compared with normative data, especially for physical aspects. This is based on the fact that although the majority of children recover rapidly with regard to functional health, a number of children are seriously impaired in physical functioning [11]. Furthermore, higher self-reported HRQoL regarding physical aspects and lower HRQoL regarding mental aspects were expected for parents’ HRQoL, based on a previous study that examined parents HRQoL after PICU admission of their child [12]. The second aim was to investigate the relation between parent-reports regarding their own HRQoL and regarding their child. It was hypothesized to find an association between the parent-reported HRQoL of the child and the self-reported quality of life of parents, since reduced parental physical and psychosocial wellbeing predicts poorer functioning of the child [3]. The third aim was to explore which baseline variables are associated with HRQoL outcomes of the child. Younger age and greater severity of illness were expected to be associated with parent-reported HRQoL outcomes of the child [2]. Overall, a lower HRQoL for patients and their parents compared to the general population was expected on this relatively short-term after PICU admission.

## Methods

### Participants and procedure

This study included critically ill children who participated in the Pediatric Early versus Late Parenteral Nutrition in Intensive Care Unit (PEPaNIC) randomized controlled trial (RCT). All children (term newborns—18 years old) who were admitted to one of the participating pediatric ICUs (University Hospitals Leuven, Belgium; Erasmus MC–Sophia Children’s Hospital, the Netherlands, and Stollery Children’s Hospital Edmonton, Canada) were eligible for inclusion in the PEPaNIC RCT if a stay of 24 h or more in the ICU was expected. The extensive trial protocol and medical outcomes of this RCT have been published previously [13, 14]. The institutional review board at each of the 3 participating sites approved the protocol (ML8052; NL49708.078; Pro00038098). The PEPaNIC study enrolled 1440 children who were admitted to the PICU. Participating children were randomly assigned to early (within 24 h) or late (not in the first week) supplementation of insufficient enteral nutrition with parenteral nutrition. The current study is a secondary analysis of cross-sectional data collected in a group of children who participated in the PEPaNIC trial.

At inclusion in the PEPaNIC study, parents had given informed consent for inviting them later for participation in a follow-up with HRQoL questionnaires. Due to logistical reasons only data of children from Belgium and The Netherlands were used in the present study. Participation in the original RCT had ended for the children at the moment they were discharged from the PICU. Six months after PICU discharge, all 1343 children included in the PEPaNIC study in Belgium and the Netherlands were screened for survival status via use of hospital notes, National Registers, and/or contact with the general practitioner or referring pediatrician. After this screening, parents of surviving children were sent HRQoL the questionnaires at home or through email. One of the parents completed the questionnaires. It was unclear whether this was the mother or the father. Results of these questionnaires are presented in this paper.

### Instruments

Three internationally validated questionnaires with satisfactory psychometric characteristics were used to measure HRQoL. A higher score reflects better HRQoL for all questionnaires.

Parents of patients 0–3 years old completed the Infant–Toddler Quality of Life Questionnaire (ITQOL) about HRQoL of their child [15]. The ITQOL consists of 103 items on 12 scales of HRQoL (for a description of the scales see Online Resource 1). Two scales (“General behavior” and “Getting along”) are only relevant for parents of children older than 1 year. The ITQOL has a good internal consistency (Cronbach’s alpha > 0.70). Test–retest intraclass correlation coefficients were moderate or adequate (≥ 0.50; *p* < 0.001) [16]. Parents of patients 4–18 years old completed the Child Health Questionnaire-Parent Form 50 (CHQ-PF50) about HRQoL of their child [17]. The CHQ-PF50 consists of 50 items on 13 scales of HRQoL (for a description of the scales see Online Resource 1). The internal consistency for the CHQ-PF50 is good, with Cronbach’s alpha for Dutch school children ranging from 0.39 to 0.96 for an average of 0.72 for the subscales [18]. The ITQOL and CHQ-PF50 are parallel forms of the same questionnaire, adapted to the age of the child. This means that nine scales of the questionnaires overlap: physical functioning, bodily pain, general behavior, general health perceptions, parental impact: emotional, parental impact: time, family activities, family cohesion, and change in health (for additional scales of the two forms see Online Resource 1, Tables 2, 3).

Parents completed the Short Form Health Survey (SF-12) regarding their own HRQoL. The SF-12 is a short version of the SF-36 which has shown to be an adequate reproduction with a lower burden for the responder. The SF-12 consists of 12 items [19, 20]. The “Physical Component Summary” (PCS) and the “Mental Component Summary” (MCS) are reported. The internal consistency of the SF-12 is good, with Cronbach’s alpha coefficients of 0.72 to 0.89. Test–retest reliability ranged between 0.73 and 0.86 [21].

For the third aim of the study, regarding the variables associated with HRQoL outcomes of the child, baseline characteristics were collected during admission to the PICU. The baseline variables collected during PICU admission were age at admission, gender, reason for admission (urgent or elective), length of stay, PIM2 (pediatric index of mortality), PELOD (pediatric logistic organ dysfunction), and diagnosis (cardiac surgery, surgery other, neurological, medical other). PIM2 and PELOD scores give an indication of severity of illness.

### Norm groups

The Dutch version of HRQoL measurements was used in both Belgium and the Netherlands. Results of both groups were compared using Dutch normative data, since available Belgian normative data consisted of small norm groups. The Dutch norm group of the ITQOL included parents of 410 children [16]. For the CHQ-PF50, the norm group consisted of 353 parents of Dutch school-aged children. No Dutch norms are available for the subscale “Change in health” of the CHQ-PF50 [18]. For the SF-12, the norm group consisted of 2301 adults from the general Dutch population [20].

### Statistical analysis

Baseline demographics and clinical variables for surviving children with and without follow-up data at 6 months were compared with Mann–Whitney *U* tests (continuous data) or *χ*^2^ tests (discrete data). Baseline continuous demographical and clinical variables were summarized using median and interquartile range (IQR). Discrete variables were summarized as count and percentage. Following the scoring instructions of the instruments, the scale item scores for the ITQOL and CHQ-PF50 were summed and transformed into 0 (worst possible health state) to 100 (best possible health state) scale scores. Some specific items were recoded to ensure that all items were positively scored and that higher scores indicated better health. Items with an “excellent to poor” response continuum were recalibrated to achieve a better linear fit with corresponding scales and to provide a better estimation of equal interval scaling [22]. The SF-12 “Physical Component Summary” and the SF-12 “Mental Component Summary” were transformed into *T*-scores (mean 50, standard deviation 10). The internal consistency of the ITQoL scales, CHQ-PF50 scales, and SF-12 scales with 2 or more items per scale were calculated with Cronbach’s alpha. Chronbach’s alphas > 0.70 were considered good. Mean scale scores of the ITQOL, CHQ-PF50, and SF-12 scores were compared with normative data using Student’s t tests and were reported as means and standard deviation. Cohen’s *d* effect sizes were calculated by determining the mean difference between the two groups and subsequently dividing this difference by the pooled standard deviation. Effect sizes of smaller than 0.5 were considered small, effect sizes between 0.5 and 0.8 were considered medium and effect sizes greater than 0.8 were considered large. The associations between parents’ reports regarding HRQoL of their child and of themselves were analyzed using Pearson Correlations. Correlations of lower than 0.30 (positive or negative) were considered weak, correlations between 0.30 and 0.70 (positive or negative) were considered moderate, and higher than 0.70 (positive or negative) were considered strong.

To assess which baseline variables are associated with subscales of parent-reported HRQoL outcomes of the child, overlapping scales of the ITQOL-97 and CHQ-PF50 were combined to have one score on those scales across all ages. In linear regression analyses (univariate analyses), each baseline variable was associated with each overlapping subscale. When the association had a significance of *p* < 0.10, the variable was inserted into the multiple regression analysis. After multiple regression analysis, baseline variables with *p* < 0.10 were included in the final model, and variables with *p* ≥ 0.10 were removed (backward elimination procedure). For the association between the remaining variables and the HRQoL subscale, the total explained variance (R2) was calculated.

## Results

### Baseline characteristics of the critically ill children

**Fig. 1:**
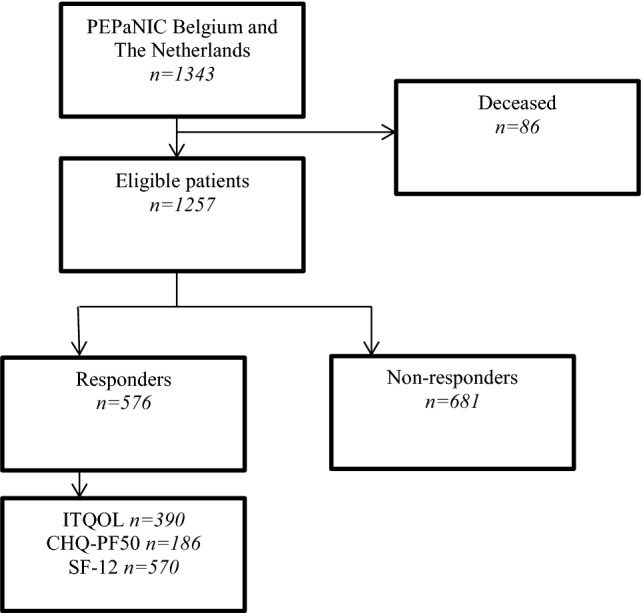
Flowchart of inclusion. *ITQOL* infant and toddler quality of life questionnaire, *CHQ*-*PF* child, health questionnaire-parent form, *SF* short form health survey

Of the total patient population (*N* = 1343), 86 (6%) children had died within 90 days after PICU admission. Parents of 576 surviving children (46%, 278 of the early parenteral nutrition group and 298 of the late parenteral nutrition group) completed the questionnaires (Fig. 1). Although the original PEPaNIC study is a randomized controlled trial, too many patients did not have follow-up data at 6 months (343 of the early parenteral nutrition group, 338 of the late parenteral nutrition group) to compare randomization groups. Therefore, the analyses were conducted on the complete group of critically ill children who participated in this short-term follow-up assessment. Children of responding parents had less often an acute reason for admission than an elective reason for admission, and they differed in type of diagnosis, compared with children of non-responders. When children of both age groups were compared (children aged 0–3 years and children aged 4–18 years), differences were found in age, but also in risk of mortality (lower risk in older children) and diagnosis (Table 1).

**Table 1:** Baseline characteristics of children from parents who responded and from parents who not responded

Characteristic	Responders (*n *= 576)		Non-responders (*n *= 681)		*p*-value	Children aged 0–3 years (*n *= 390)		Children aged 4–18 years (*n *= 186)		*p*-value
Child characteristics										
Age in years at admission	1.3	(0.2 to 5.6)	1.5	(0.3 to 7.4)	0.09	0.4	(0.1 to 1.5)	8.8	(5.8 to 13.6)	
Gender (male)	330	57.3%	403	59.2%	0.50	229	58.7%	101	54.3%	0.32
Disease characteristics										
Acute admission	269	46.7%	307	45.1%	**< 0.01**	187	47.9%	82	44.1%	0.39
Length of stay	3.0	(2.0 to 7.0)	4.0	(2.0 to 7.0)	0.63	4.0	(2.0 to 7.3)	2.0	(1.0 to 5.0)	0.79
PIM2	− 3.0	(− 3.7 to − 1.9)	− 2.8	(− 3.7 to − 1.7)	0.31	− 2.9	(− 3.6 to − 1.7)	− 3.1	(− 3.8 to − 2.1)	**< 0.01**
PELOD	21.0	(12.0 to 31.0)	21.0	(11.0 to 31.0)	0.05	21.0	(12.0 to 31.0)	21.0	(11.0 to 31.0)	0.15
Diagnosis					**< 0.01**					**< 0.01**
Cardiac surgery	264	45.8%	239	35.1%		189	48.5%	75	40.3%	
Surgery other	179	31.1%	222	32.6%		103	26.4%	76	40.9%	
Neurological	30	5.2%	58	8.5%		23	5.9%	7	3.8%	
Medical other	103	17.9%	162	23.8%		75	19.2%	28	15.1%	

Data are presented as number of subjects (%) in the group, except for age, length of stay, PIM2 (Pediatric Index of Mortality 2), and PELOD (Pediatric Logistic Organ Dysfunction) which are presented as median (interquartile range). *p*-values were considered statistically significant with two-tailed *p*-values of less than 0.05 in which case they are expressed in bold. PIM2 estimates mortalitiy risk (higher score means less probability of mortality, less severe illness), PELOD describes the severity of organ dysfunction (higher score means more severe illness). Diagnostic group was determined by diagnosis at admission and was classified in the following way: cardiac surgery: cardiac surgery, surgery other: abdominal, burns, neurosurgery, thoracic, transplantation, orthopedic surgery-trauma, and other surgery, neurological: medical neurologic, medical other: cardiac medical, gastrointestinal-hepatic, oncologic-hematologic, neurologic, renal, respiratory and medical other [16]

### HRQoL of critically ill children 6 months after PICU admission

As to the internal consistency of the instruments in the current sample, the Cronbach’s alphas of the ITQoL scales averaged 0.87 (0.74–0.94), those of the CHQ-PF50 scales averaged 0.85 (0.66–0.98), and those of the SF-12 averaged 0.86 (0.75–0.93). Only the CHQ-PF50 scale ‘mental health’ (*α* = 0.66) had a Cronbach’s alpha < 0.70.

Parents of 390 children between 0-3 years old (44%) completed the ITQOL (Table 2). HRQoL of PICU children (0–3 years old) was lower compared with normative data on “Physical functioning”, “Growth and development”, “Bodily pain”, “Temperament and moods”, “General health perceptions”, “Parental impact” (Emotional and Time), and “Family activities” (*p *< 0.001). Scores were comparable on “General behavior” (*p *= 0.130) and “Getting along” (*p *= 0.936), which were completed only by parents of children older than 1 year. Parent-reported HRQoL of the child was higher than normative data on “Family cohesion” and “Change in health” (*p *< 0.001). Effect sizes were medium to large except for two scales (“Temperament and moods”, and “Family cohesion”) on which effect sizes were small.

**Table 2:** Parent-reported mean scores of Infant–Toddler quality of life and Child Health Questionnaire-Parent Form 50

Subscale	Infant–Toddler Quality of Life (ITQOL; 0–3 years)					Child Health Questionnaire-Parent Form (CHQ-PF50; 4–18 years)				
	*n*	Patients	Norm	*p*-value patients versus norm	Effect size Cohen’s *d*	*n*	Patients	Norm	*p*-value patients versus norm	Effect size Cohen’s *d*
Physical functioning	354	84.8 (22.6)	97.2 (9.8)	**< 0.01**	0.71	181	73.9 (32.5)	99.1 (4.3)	**< 0.01**	1.08
Growth and development	390	79.5 (15.7)	86.5 (10.6)	**< 0.01**	0.52					
Bodily pain	389	72.8 (22.6)	83.8 (16.8)	**< 0.01**	0.55	183	71.5 (25.4)	85.7 (17.2)	**< 0.01**	0.65
Temperament and moods	386	74.8 (12.8)	77.2 (10.5)	**< 0.01**	0.20					
General behavior^a^	159	74.6 (15.1)	72.8 (12.7)	0.13	0.12	181	75.2 (15.1)	78.5 (13.1)	**< 0.01**	0.23
Getting along^a^	158	71.5 (10.2)	71.4 (8.8)	0.94	0.01					
General health perceptions	386	51.3 (20.4)	79.0 (14.5)	**< 0.01**	1.56	183	51.3 (24.6)	82.9 (13.4)	**< 0.01**	1.59
Parental impact: emotional	387	80.9 (19.9)	92.1 (10.5)	**< 0.01**	0.70	184	62.5 (30.6)	86.3 (15.2)	**< 0.01**	0.98
Parental impact: time	386	80.8 (21.0)	93.0 (11.0)	**< 0.01**	0.72	184	73.6 (31.2)	94.0 (13.0)	**< 0.01**	0.85
Family activities	385	71.2 (24.6)	86.2 (13.5)	**< 0.01**	0.75	183	72.5 (25.9)	91.5 (11.9)	**< 0.01**	0.94
Family cohesion	385	79.1 (17.9)	75.3 (18.8)	**< 0.01**	0.20	183	72.4 (21.6)	72.2 (19.4)	0.90	0.01
Change in health^a^	119	70.0 (30.5)	56.1 (18.4)	**< 0.01**	0.55					
Role functioning emotional/behavioral						179	79.0 (33.8)	97.9 (7.2)	**< 0.01**	0.77
Role functioning physical						175	71.0 (37.3)	95.8 (15.6)	**< 0.01**	0.86
Mental health						181	73.0 (14.3)	81.4 (12.1)	**< 0.01**	0.63
Self-esteem						175	73.2 (17.7)	79.2 (11.0)	**< 0.01**	0.40

Data are presented as means (standard deviation). A higher score represents a better HRQoL (0 is worst possible health state, 100 is best possible health state). *p*-values were considered statistically significant with two-tailed *p*-values of less than 0.05 in which case they are expressed in bold. Cohen’s *d* effect sizes of < 0.5 were considered small, < 0.8 medium and > 0.8 large

^a^Scales are only applicable to children aged 1 year or older. For a description of the subscales see Online Resource 1. “Change in health” was assessed in the CHQ-PF 50 as well, but no Dutch normative data are available which explains why this scale was not analyzed. Some parents did not complete all questions of a scale which resulted in differences between sample sizes on the subscales

Parents of 186 children between 4 and 18 years old (42%) completed the CHQ-PF50 (Table 2). Parent-reported HRQoL of these children was lower compared with normative data on all scales, except for “Family cohesion” (*p *= 0.898). Effect sizes were medium to large except for two scales (“General behavior” and “Self-esteem”) for which effect sizes were small.

### HRQoL of the parents

Parents of 570 children (42%) completed the SF-12 about their own HRQoL. Parents reported significantly higher scores than normative data on the “Physical component summary” of their own HRQoL (*n *= 555, parents of patients 53.7 (SD 7.6) versus norm 50.7 (SD 9.2), *p* < 0.001, Cohen’s *d* 0.35). The effect size was small. Parents scored significantly lower than normative data on the “Mental component summary” of their own HRQoL (*n *= 556, parents of patients 47.2 (SD 12.1) versus norm 50.5 (SD 9.4), *p *< 0.001, Cohen’s *d* .30). Also here, the effect size was small.

### Associations between HRQoL of the parents and that of their children

No significant correlations were found between the self-reported SF-12 “Physical component summary” and the scales of the parent-reported ITQOL and CHQ-PF50 regarding the child’s HRQoL, except for “Physical functioning” (ITQOL, Pearson Correlation 0.12, *p *= 0.028) and “Bodily pain” (CHQ-PF50, Pearson Correlation .18, *p *= 0.021) (Table 3). The self-reported SF-12 “Mental component summary” significantly correlated with all scales of the parent-reported ITQOL and CHQ-PF50 regarding the child’s HRQoL (Pearson Correlations ranges 0.25–0.57, *p *< 0.001–0.002) (Table 3). These correlations are all positive, which means that when the score on the self-reported SF-12 “Mental component summary” was higher, scores on scales of the parent-reported ITQOL and CHQ-PF50 for the child were also higher. Regarding the strengths of the correlations, most scales of the child’s HRQoL were moderately correlated to the mental component of parents HRQoL (Table 3).

**Table 3:** Correlations between parent-reported quality of life in children (ITQOL; 0–3 years and CHQ-PF50; 4–18 years) and their own quality of life (SF-12)

Subscale	ITQoL				CHQ-PF50			
	SF-12 Physical component summary		SF-12 Mental component summary		SF-12 Physical component summary		SF-12 Mental component summary	
	Pearson correlation	*p*-value	Pearson correlation	*p*-value	Pearson correlation	*p*-value	Pearson correlation	*p*-value
Physical functioning	0.12	**0.028**	0.29	**< 0.01**	0.06	0.455	0.32	**< 0.01**
Growth and development	0.02	0.635	0.41	**< 0.01**				
Bodily pain	0.02	0.665	0.37	**< 0.01**	0.18	**0.021**	0.25	**< 0.01**
Temperament and moods	0.07	0.192	0.37	**< 0.01**				
General behavior	0.14	0.083	0.25	**< 0.01**	0.06	0.417	0.28	**< 0.01**
Getting along	0.11	0.174	0.27	**< 0.01**				
General health perceptions	0.04	0.498	0.54	**< 0.01**	0.13	0.089	0.36	**< 0.01**
Parental impact emotional	0.03	0.566	0.50	**< 0.01**	0.09	0.221	0.53	**< 0.01**
Parental impact time	0.05	0.309	0.50	**< 0.01**	0.12	0.117	0.50	**< 0.01**
Family activities	− 0.004	0.933	0.57	**< 0.01**	0.12	0.102	0.54	**< 0.01**
Family cohesion	− 0.02	0.744	0.31	**< 0.01**	0.11	0.145	0.36	**< 0.01**
Change in health	− 0.13	0.181	0.35	**< 0.01**	0.06	0.402	0.27	**< 0.01**
Role functioning emotional/behavior					0.15	0.050	0.39	**< 0.01**
Role functioning physical					0.02	0.833	0.42	**< 0.01**
Mental health					0.11	0.154	0.43	**< 0.01**
Self-esteem					0.03	0.709	0.39	**< 0.01**

Correlations were considered statistically significant with two-tailed *p*-values lower than .05 (expressed in bold)

### Baseline PICU variables associated with 6 months’ HRQoL of the child

Baseline variables during PICU stay explained the most variance in the following scales (ranging from 12 to 26%): parent-reported physical functioning of the child, change in health of the child, and parental impact emotional, compared with the other six parent-reported HRQoL scales of the child (explained variances lower than 10%) (Online Resource 2 and Table 4). Higher age at admission of the child, longer length of PICU stay, a higher PIM2 score (higher risk of mortality), and other diagnoses than cardiac surgery were associated with worse scores for children on parent-reported physical functioning, change in health, and parental impact emotional. Overall as to diagnosis, parents of children with cardiac surgery reported the most favorable scores and parents of children with a neurological diagnosis reported the lowest scores on physical functioning, change in health, and parental impact emotional.

**Table 4:** Final model results of baseline characteristics associated with overlapping scales of the infant–Toddler Quality of Life Questionnaire (ITQOL) and Child Health Questionnaire-Parent Form 50 (CHQ-PF50)

Subscale	*n*	Constant	Unstandardized *β*	SE	Standardized *β*	*p*-value	Multiple *R*^2^
**Impact on the child**							
Physical functioning	535						
Age at admission in years		83.02	− 1.13	0.23	− 0.20	< 0.01	0.12
Length of stay			− 0.33	0.12	− 0.12	< 0.01	
PIM2			− 3.10	0.83	− 0.17	< 0.01	
Diagnosis—surgery other^a^			− 9.66	2.66	− 0.17	< 0.01	
Diagnosis- neurological^a^			− 15.66	5.04	− 0.13	< 0.01	
Diagnosis—medical other^a^			− 1.67	3.10	− 0.02	0.59	
Bodily pain	572						
Length of stay		69.47	− 0.34	0.11	− 0.14	< 0.01	0.05
PIM2			− 1.86	0.68	− 0.12	0.01	
General behavior							
Diagnosis—surgery other^a^		75.01	− 0.00	1.86	0.00	0.99	0.02
Diagnosis- neurological^a^			− 7.26	3.67	− 0.11	0.05	
Diagnosis—medical other^a^			1.87	2.34	0.05	0.42	
General health perceptions	569						
Length of stay		49.87	− 0.27	0.10	− 0.12	< 0.01	0.06
PIM2			− 1.75	0.67	− 0.12	< 0.01	
Diagnosis—surgery other^a^			− 1.00	2.15	− 0.02	0.64	
Diagnosis—neurological^a^			− 6.02	4.16	− 0.06	0.15	
Diagnosis—medical other^a^			− 5.72	2.52	− 0.10	0.02	
Change in health	300						
Age at admission in years		89.85	− 1.12	0.37	− 0.16	< 0.01	0.26
Reason for admission^b^			− 13.43	5.36	− 0.19	0.01	
Length of stay			− 0.43	0.18	− 0.12	0.02	
Diagnosis—surgery other^a^			− 16.39	4.72	− 0.23	< 0.01	
Diagnosis—neurological^a^			− 23.62	9.18	− 0.16	0.01	
Diagnosis—medical other^a^			− 20.92	7.05	− 0.23	< 0.01	
**Impact on the family**							
Parental impact emotional	571						
Age at admission in years		81.35	− 1.78	.21	− 0.33	< 0.01	0.18
Length of stay			− 0.23	0.11	− 0.09	0.03	
PIM2			− 2.14	0.73	− 0.13	< 0.01	
Diagnosis—surgery other^a^			− 10.11	2.37	− 0.18	< 0.01	
Diagnosis—neurological^a^			− 11.95	4.60	− 0.10	0.01	
Diagnosis—medical other^a^			− 3.36	2.74	− 0.05	0.22	
Parental impact time	570						
Age at admission in years		75.30	− 0.85	0.22	− 0.16	< 0.01	0.09
Gender^c^			4.53	2.05	0.09	0.03	
PIM2			− 3.01	0.76	− 0.18	< 0.01	
Diagnosis—surgery other^a^			− 8.87	2.46	− 0.16	< 0.01	
Diagnosis—neurological^a^			− 9.73	4.86	− 0.08	0.05	
Diagnosis—medical other^a^			0.20	2.84	0.00	0.95	
Family cohesion	568						
Age at admission in years		79.97	− 0.83	0.17	− 0.20	< 0.01	0.04
Family activity	568						
Length of stay		69.75	− 0.36	0.11	− 0.14	< 0.01	0.04
PIM2			− 1.53	0.73	− 0.09	0.04	

^a^Reference category is diagnosis cardiac surgery

^b^Elective = 0, acute = 1

^c^Male = 0, female = 1

## Discussion

Overall, HRQoL of children 6 months after critical illness, as reported by parents, appeared to be lower than that of healthy peers of the general population. With regard to parents’ own HRQoL, parents reported higher scores on physical aspects of HRQoL and lower scores on mental aspects of HRQoL compared with adults from the general population. Furthermore, parents’ own mental HRQoL showed positive associations with scales of HRQoL that they reported for their child.

In line with previous research, parent-reported HRQoL of children in the short-term after critical illness was lower compared with healthy children [7, 8]. In the current study, most domains were impaired in PICU survivors as reported by the parent. However, on a few scales PICU survivors scored comparable or even better than healthy children. One of these scales is the family cohesion, indicating that the relationships between family members did not seem to be impaired. Therefore, although critical illness of a child impacts the emotional state of each family member [23], it does not seem to impact the bonds within the family. Moreover, it may even strengthen bonds as is reflected in results of the ITQOL in the current study. This might probably be due to enhanced awareness of the value of these relationships in burdensome times, shortly after critical illness of the child. This could be a result of a response shift, in which parents value certain aspects of life more as a consequence of the difficult situation they are in [3, 24]. It has also been reported that strengths of attachment within the family increase in the short-term after PICU admission of a child [23].

With regard to behavioral aspects of HRQoL, parents of older PICU survivors (4–18 years) reported worse scores for their children compared to healthy peers on the subscale ‘general behavior’. This is in contrast with a study that used the same questionnaire and that examined HRQoL in school-aged children 10 years after admission to the PICU for meningococcal disease [12]. Children did not show significant differences with normative data on the subscale ‘general behavior’ in this study. Possibly, the longer follow-up interval compared to the current study caused the differences between the two studies. This suggests that children on the short-term have to adjust their behavior, but show behavior that is similar to their peers on the longer term.

### The role of parents’ own HRQoL in their reports of HRQoL of their children

Parents reported that their own HRQoL with regard to physical aspects was better than that of adults from the general population. In a previous study, parents reported that the PICU admission of their child made them appreciate life more fully [12]. Especially when parents have seen the possible poor physical health state a person can be in, their internal standards of physical health may change. This is called a response shift [24].

With regard to mental aspects of HRQoL, parents reported worse HRQoL for themselves compared with adults from the general population. This appears to reflect the psychosocial burden of critical illness of their child and is in line with previous studies [3, 25]. These psychosocial symptoms are common among parents of children previously admitted to the PICU [26, 27]. In the short-term, 6 months after admission, parents have to adjust to the psychosocial burden they experienced due to the critical illness in their child.

Parents’ own mental HRQoL was positively correlated to the HRQoL they report for their child. This means that when parents’ mental HRQoL is better, parent-reported HRQoL of the child is also better. The association between parent-reported HRQoL of children and self-reported HRQoL of parents themselves might be explained by the fact that family characteristics influence children’s HRQoL [3]. When parents experience impairments in their mental health as an effect of the PICU admission of their child, this will influence the way the family is functioning. Since the child is dependent on the parents for physical, emotional. and social needs, their HRQoL will be lower as well [3]. However, the found association between the HRQoL of the child and the HRQoL of the parents could also be reflection of the distress that parents experience [24]. This might also explain why parents in previous studies report more problems than the child regarding the child’s health status [28]. The phenomenon of these differences between parent-reports and self-reports of the child’s HRQoL is called the proxy-problem and has been extensively studied [29].

### Variables during PICU stay associated with HRQoL outcomes

Parents of children who were admitted to the PICU report worse scores for physical functioning and change in health of their child when the child had a higher age at admission, had a longer length of stay and had a more severe illness. Furthermore, when the diagnosis of the child was related to a cardiac surgery, parent-reported physical functioning and change of health was higher for the child. With regard to the emotional impact on the parent we found the same variables that were associated with lower HRQoL outcomes of the child. The associations between length of stay and severity of illness with parent-reported HRQoL of the child are in line with results that have been found in previous reviews [2, 23, 30]. Age and diagnosis are relatively less studied in these reviews. However, it should be noted that the sample size of children with a neurological condition in the current study was relatively low, which could have influenced the results.

### Implications

Considering the impaired HRQoL of children a few months after PICU admission, identifying children most at risk by asking parents to complete HRQoL questionnaires should be part of the follow-up care to intervene early and to prevent problems on the longer term. The self-reported mental wellbeing of parents, which was associated with their reports on HRQoL outcomes for children, suggests that the focus of follow-up interventions might have to involve the entire family. Furthermore, HRQoL outcomes of critically ill children and the impact of parents’ own perceived HRQoL in the longer term after critical illness of the child could be investigated. A study that examined the longer term in the most critically ill children (who needed a prolonged PICU stay) with a mean follow-up of 6 years showed that although some children recover from the HRQoL impairments, almost half of the children were at risk for impaired HRQoL on the longer term [31], what suggests that research into children who experience impaired HRQoL on the longer term is necessary. Lastly, since a higher age at admission, a longer length of PICU stay, a more severe illness, and another diagnosis than cardiac diagnosis were associated with lower HRQoL of the child, children and parents with these characteristics are of special attention in follow-up programs.

### Limitations and strengths

Our study has some limitations that need to be addressed. First, despite the large sample size, the response rate was relatively low. However, other follow-up studies that examined HRQoL on the short-term after critical illness showed similar response rates [7, 32]. Due to the relatively low response rates, we decided not to analyze the effects of the RCT, which is a shortcoming of the study as well since withholding parenteral nutrition during the first week of critical illness might have influenced the HRQoL outcomes in a beneficial way as the short-term medical outcomes were positive as well [13]. Another limitation of this study is that no self-reports of children regarding their own HRQoL were reported. Although proxy-reports are valuable instruments since parents are so closely involved in the child’s life [24], some scales are subjective and might be hard to observe by the parents [29], such as mental health and self-esteem. However, only 12% of the children in our sample was old enough (12 years or older) to be able to report their own HRQoL with the self-report version of the questionnaire. Therefore, proxy-reports were unavoidable. Nevertheless, the current results should be interpreted from the perspective of the parent, and therefore with caution. Furthermore, children of responding parents differed in emergency of admission and diagnosis, compared with children of non-responding parents. Lastly, all data of Dutch and Belgian children and parents were compared to Dutch normative data. Differences might exist between the Dutch and Belgian general population. The Dutch normative data show little differences compared to the sample of children in the current study regarding gender (ITQOL norm data 50% girls [16], study sample 41% girls, CHQ-PF50 norm data 54% girls [18], study sample 46% girls) and age (ITQOL norm data mean 2.1 years [16], study sample mean 0.9 years, CHQ-PF50 norm data mean 8.8 years [18], study sample mean 9.5 years). In the current sample there are a bit more girls and the children were a little younger. The sample-based internal consistency of the HRQoL instruments used were satisfying and were comparable or even better than the internal consistency as reported for the normative groups in the concerning manuals. However, the current results should be generalized with caution.

A strength of the current study is that this study is unique in its sample size, which is much larger than most studies on HRQoL of PICU survivors. The added value of this study is that it not only examined HRQoL of children and parents after PICU admission, but also investing ated the relation between parent-reports regarding their own HRQoL and regarding their child.

## Conclusion

HRQoL seems to be important in evaluating the health status of critically ill children and is usually reported by parents. Six months after discharge from the PICU this HRQoL of the child is lower compared with healthy children from the general population. The current study suggests that parents’ own physical health after PICU admission of their child is better than that of the general population of adults, but that their mental HRQoL is lower. These lower scores on mental health of parents seem to be associated with lower HRQoL they report for their children. Therefore, parents should also be targeted in follow-up care for PICU survivors, but more research on this parental role is needed.

## Electronic supplementary material

Below is the link to the electronic supplementary material.

Supplementary material 1 (DOCX 21 kb)

Supplementary material 2 (DOCX 17 kb)
