# Supplementary material for: Health-related quality of life of children and their parents 6 months after children’s critical illness
Source: Qual Life Res. 2019 Nov 6;29(1):179–89. doi: 10.1007/s11136-019-02347-x (PMC6962289; doi:10.1007/s11136-019-02347-x)
Supplement: Supplementary file 2 — Supplementary material 2 (DOCX 17 kb) [file 11136_2019_2347_MOESM2_ESM.docx]

| Supplemental table Associations between baseline characteristics and overlapping scales of the Infant Toddler Quality of Life Questionnaire (ITQOL) and Child Health Questionnaire – Parent Form 50 (CHQ-PF50); standardized coefficients β | | | | | | | | | | |
| --- | --- | --- | --- | --- | --- | --- | --- | --- | --- | --- |
|  |  | Age at admission in years | Gender^a^ | Reason for admission^b^ | Length of stay | PIM2 | PELOD | Diagnosis  Surgery other^c^ | Neurological^c^ | Medical other^c^ |
| Subscale | *n* |  |  |  |  |  |  |  |  |  |
| Physical functioning | 535 | -.21* | .06 | -.002 | -.18* | -.15* | .02 | -.12* | -.10* | -.00 |
| Bodily pain | 572 | -.01 | .03 | -.02 | -.18* | -.17* | -.06 | -.02 | -.04 | -.08* |
| General behavior | 340 | .08 | -.002 | .02 | .07 | -.06 | -.03 | .00 | -.12* | .06 |
| General health perceptions | 569 | -.02 | .001 | .02 | -.17* | -.18* | -.02 | .06 | -.05 | -.14* |
| Change in health | 300 | -.20* | .03 | -.42* | -.23* | -.11* | .14* | -.17* | -.14* | -.25* |
| Parental impact emotional | 571 | -.35* | .01 | -.07* | -.13* | -.08* | .007 | -.17* | -.07* | -.01 |
| Parental impact time | 570 | -.17* | .08* | -.05 | -.11* | -.12* | -.02 | -.12* | -.06 | .02 |
| Family cohesion | 568 | -.20* | -.02 | .01 | -.05 | -.01 | -.01 | -.02 | -.03 | -.02 |
| Family activity | 568 | .01 | .05 | -.02 | -.17* | -.14* | -.03 | -.03 | -.06 | -.06 |
| ^a^ Male = 0, female = 1  ^b^ Elective = 0, acute = 1  ^c^ Reference category is diagnosis cardiac surgery  * *p* <. 10, and thus included in the multiple regression analysis | | | | | | | | | | |
